# A novel questionnaire for evaluating digital tool use (DTUQ-D) among individuals with type 2 diabetes: exploring the digital landscape

**DOI:** 10.3389/fpubh.2024.1374848

**Published:** 2024-04-05

**Authors:** Ora Peleg, Efrat Hadar, Meyran Boniel-Nissim

**Affiliations:** Max Stern Academic College of Emek Yezreel, Emek Yezreel, Israel

**Keywords:** digital tools, questionnaire, type 2 diabetes, eHealth, DTUQ-D

## Abstract

**Introduction:**

Effective healthcare currently incorporates a patient-centric system and accessible technology for patient self-management. This study aimed to develop and validate a novel questionnaire titled the Digital Tool Use Questionnaire for Diabetes (DTUQ-D) - a screening tool identifying the type, number, and frequency of digital tools used by Type 2 Diabetes Mellitus (T2DM) patients with within HMOs, online, and via applications.

**Methods:**

The questionnaire was administered to two ethnic groups and both genders. A mixed-methods approach was used. In the qualitative phase, the questionnaire was developed through phone surveys of 29 T2DM patients, two endocrinologists and two technology experts. In the quantitative phase, involving 367 participants, convergent validity, construct validity, and reliability were examined.

**Results:**

Findings indicated that the DTUQ-D is valid and reliable, successfully identifying digital tools utilized by T2DM patients, notwithstanding variations in factor structures between ethnic groups. This questionnaire provides a foundation for future research, offering a standardized approach to evaluating digital tool usage.

**Discussion:**

The study enhances understanding of the role of digital tools in healthcare, especially for T2DM self-management. It also can be easily adapted to assess digital tool use for other illnesses by adjusting instructions and the wording of certain items

## Introduction

Today’s healthcare system faces such challenges as remote patient residence and costly long-term treatment of chronic diseases tied to increased life expectancy (1). These obstacles may hinder effective disease management due to challenges in accessing suitable care. Currently, healthcare improvement opportunities include a patient-centric system (i.e., Human-Centered Design), accessible technology for a broader population, and smart tools identifying patient needs (2).

Electronic health (eHealth), a term coined in the 21st century to describe electronic information and communication technology in the health sector (3), offers an efficient and updated solution to healthcare challenges. It holds the potential to improve healthcare on local, regional, and national levels by making it more accessible (4). eHealth was found to be related to health literacy (knowledge of how to obtain and use health-related information to inform health-related decisions), as higher health literacy was linked to elevated eHealth literacy (5). This suggests that individuals with strong eHealth literacy possess the skills to navigate and utilize online health information effectively (6).

For individuals with Type 2 Diabetes Mellitus (T2DM), various digital tools have been proven effective for self-management of the illness (7, 8). In light of the multitude of tools from different sources (HMO, apps, online) on the one hand, and the value of understanding precisely which digital uses actually promote healthy management of *T2DM*, on the other hand, there is a need to investigate what digital tools people with diabetes prefer to use.

To address this issue, we aimed to develop and validate a novel questionnaire – the Digital Tool Use Questionnaire for Diabetes (DTUQ-D) – a screening instrument that explores the usage of digital tools provided to T2DM patients to enhance their successful self-management. Considering the differences between the two major ethnic groups in Israel, including variations in the use of digital tools and the prevalence of T2DM, the study was conducted among Israeli Jewish patients (the majority group) and Israeli Arab patients (the minority group), including both genders. It is our hope that the data collected through this instrument will serve as a valuable resource for planning interventions aimed at optimizing the use of digital technology while mitigating potential challenges.

### T2DM self-management, eHealth literacy, and digital tool use

T2DM is a global epidemic, with the number of patients constantly rising (approximately 422 million) (9). In Israel, according to the International Diabetes Federation the age-standardized diabetes rate, for those aged 20–79 (9.7%) is higher than the average in European countries (6.3%) (10). It is one of the major diseases that can result in premature death and medical complications (9), thus putting a heavy, often financial, burden on both the healthcare system and T2DM patients and their families. Successful self-management of the illness, such as continuous glucose monitoring, can reduce these burdens (11).

Beneficial coping with chronic diseases, such as T2DM, has been associated with eHealth, which can enhance health protection, prevent hospitalizations and premature mortality, and reduce financial costs (12). Encompassing advanced information technologies (e.g., medical information sites, digital tools) for health improvement (13), eHealth aims to increase healthcare efficiency, improve the quality of care, empower patients and their families, and foster better relationships and communication between patients and healthcare professionals (14). Consequently, eHealth presents a substantial opportunity for proactive health management. eHealth literacy, defined as the ability to seek, comprehend, and assess health information from digital sources, can foster positive attitudes to eHealth services (15), augmenting self-management for T2DM patients.

In recent years, the landscape of digital services designed for individuals with T2DM has experienced rapid development, marked by the proliferation of smartphone uses, advancements in wireless communication quality, and the emergence of numerous applications dedicated to health and fostering a healthy lifestyle. While many of these applications function as health “consultants,” an increasing number are designed to establish health goals, modify health indicators, and guide patients in their daily routines (16). Several digital tools have been found to do so successfully (17). They play a pivotal role in T2DM self-management, offering a range of benefits. Notable examples of digital tools relevant to T2DM patients include websites (e.g., diabetes associations websites) and social media (e.g., Facebook forums), providing information, and mobile apps such as nutrition apps (18), physical activity apps (19), glucose monitoring apps (20), insulin titration apps (18), digital home glucose meters (glucometers) (21), bluetooth-enabled blood glucose meters (22) and T2DM treatment AI-based algorithms (23). Integrating these tools into T2DM care holds transformative potential for managing and preventing this chronic condition (24). A recent study (25) found that registration for the use of HMO-provided digital tools, such as online medical consulting services, was associated with adherence to a medication regimen and positive treatment outcomes. These tools seem to offer real-time insights into blood glucose dynamics, trends, and indications for hypoglycemia and hyperglycemia, enhancing the safety and effectiveness of T2DM care (24). Additional advantages of digital tools are their convenience in overcoming logistical challenges, reaching populations that may avoid seeking treatment, and their relatively low cost (26).

Despite the advancements in digital tools for T2DM self-management, there remains a dearth of information regarding available applications and how often they are used. This lacuna underscores the need for more efficient and accurate assessment tools (27). To thoroughly explore the utilization of digital tools among individuals with T2DM within such a diverse landscape, it is crucial to construct and implement an evaluation instrument, notably a questionnaire. It is essential to have a standardized, reliable, and validated questionnaire to assess the type, number and frequency of such digital tools used for self-management of the illness.

### Digital tool use questionnaires for T2DM patients

To date, to the best of our knowledge, only two questionnaires are available for evaluating the use of digital tools among individuals with diabetes. The first is the Instrument for Assessing Mobile Technology Acceptability in Diabetes Self-Management (28), which was developed to gauge patients’ attitudes to and intentions to use mobile technology for diabetes self-management. This questionnaire was developed through comprehensive interviews of both patients and physicians. However, its focus is on assessing attitudes rather than the actual usage of digital tools for diabetes management. While the reported reliability of 0.7 suggests moderate response consistency, it also implies some variability. Moreover, the instrument’s development could benefit from further consideration of cultural nuances, potentially enhancing its effectiveness in diverse populations with varying cultural perspectives on technology.

The second instrument, The Diabetes Self-Management and Technology Questionnaire [DSMT-Q; (29)], is designed to assess self-management among patients with T2DM utilizing web-based and mHealth tools (i.e., mobile health, namely, health practices aided by mobile devices). The questionnaire has high reliability, with Factor 1, “Understanding individual health and making informed decisions,” comprising seven items and a Cronbach’s alpha level of 0.90. Factor 2, “Confidence to reach and sustain goals,” includes six items with a Cronbach’s alpha level of 0.88. To ensure the suitability of the DSMT-Q for T2DM patients, a preliminary study was carried out involving in-depth interviews with patients and evaluation by diabetes experts. Despite its strengths, it is essential to acknowledge that the effectiveness of this questionnaire could be enhanced by a more substantial representation of ethnic minorities in the validation sample. This would broaden the generalizability of findings to diverse populations. Moreover, considering the dynamic nature of digital tools for T2DM, regular updates to the questionnaire items and structure would ensure its continued relevance in the ever-evolving medical landscape. Finally, this questionnaire does not address the utilization of specific digital tools or the frequency of their use.

In sum, in the last decade, as far as we know, only two inventories have been developed to assess the utilization of digital tools among T2DM patients, and existing questionnaires have limitations in their scope of assessing digital tool use. To address these limitations, it is crucial to add up-to-date and well-validated tools that align with the latest digital innovations (e.g., apps for monitoring/tracking glucose, apps on a smart watch or mobile phone). Such tools should capture additional degrees and complexities of T2DM patients’ perspectives and digital tool use, while accounting for gender differences and ethnic diversity.

### Ethnic diversity

Despite the advantages of using digital tools, there is a digital divide that is characterized by differences in technological usage and accessibility to Internet infrastructure among different populations. Ethnic diversity has recently become a significant focus of T2DM research, indicating higher percentages of the illness among minority groups [e.g., (9, 30)].

In Israel, the rate of Arab T2DM patients is double that of Jewish patients (12% of Arabs and 6.2% of Jews). While the Israeli Jewish majority group is basically westernized, the Israeli Arab minority group [comprising 21% of the population; (31)] is more collectivist and is undergoing a transition from traditionalism to modernity (such as urbanization and changes in family patterns, lifestyle, and dietary habits, including increased consumption of foods rich in simple carbohydrates), such that most Arabs are considered bicultural (32, 33). Indeed, Israeli Arabs are a unique minority with affinities both to modern Israeli society and to the Arab traditional world (34). At the same time, Israeli Arabs are found to have lesser T2DM self-management and a poorer health and clinical profile than the Jewish population (35). A review of the literature highlights the high prevalence of T2DM among Arab populations in Israel, as well as the need for improved care and counseling for these populations (36).

Though considered a developed country in technological advancement and despite its strong economy, Israel is characterized by income inequality, financial gaps, and a lack of social unity between ethnic groups, leading to social and digital gaps. The digital gap between Jews and Arabs is a crucial element of Israeli society, originating from their different cultural backgrounds and aggravated by the lower socio-demographic status of Arabs (30, 32). This digital divide takes the form of differences in Internet infrastructure accessibility between these groups (37, 38). These disparities between Israeli Arabs and Jews can adversely affect T2DM self-management and exacerbate the consequences of the disease.

### The present study

For individuals with chronic conditions like T2DM, the use of eHealth tools shows promise for enhancing self-management and improving health outcomes (39). However, more research is needed to understand how these patients use digital tools, whether they use recommended tools and approaches, and how technology impacts health behaviors and health status. Limited initiatives have been undertaken to create questionnaires with which to evaluate digital tool use among patients with T2DM. In light of the lacuna of research instruments for evaluating such usage, there is a compelling need to develop a valid, reliable questionnaire that is up-to-date and culturally sensitive, incorporating technological innovations.

The current study aimed to address this gap by using a mixed-methods approach to develop (qualitative) and validate (quantitative) a novel tool, DTUQ-D. This questionnaire was specifically designed as a screening tool to evaluate the utilization (type, number, and frequency) of digital tools among diverse populations. Therefore it was posited that DTUQ-D would be associated with a similar content measure, eHealth Literacy Scale [eHEALS; (40)], supporting its validity. To this aim, the study included two phases. The first phase was qualitative with the aim of generating questionnaire items. Our research question was: what digital tools are utilized by and available to T2DM patients for self-management of their illness?

The second phase of the study was quantitative with the research question of whether the DTUQ-D is valid among the two major ethnic groups in Israel, Jewish and Arab T2DM patients, including both genders. We hypothesized that the DTUQ-D would demonstrate a positive association with the eHealth Literacy Scale among T2DM patients of both ethnic groups and genders. This instrument measures understanding and proficiency in utilizing digital medical care. Given evidence underscoring a higher incidence of T2DM among Israeli Arabs (30), we also hypothesized that Jewish T2DM patients would demonstrate higher digital tool use than their Arab counterparts. Lastly, gender differences in the research variables were examined.

## Methods

The study used a mixed-methods sequential exploratory (qualitative-quantitative) design (41). In the qualitative exploratory phase, data was collected on T2DM digital tools for DTUQ-D development. The subsequent quantitative phase involved validity, reliability, and factor analysis tests.

### Phase 1: qualitative exploratory

The first phase of the study explored the digital tools utilized by and available to T2DM patients for self-management of their illness, with the aim of generating questionnaire items. We used an inductive method of gathering information (42) about digital tools available for T2DM patients’ self-management.

#### Participants and information sources

To obtain information on relevant digital tools, we conducted a telephone survey of patients with T2DM. Inclusion criteria were diagnosis of T2DM and treatment in one of the main HMOs in Israel. Using cluster sampling, a diabetes HMO clinic in a large city in northern Israel was selected. For 2 months, patients coming for their regular checkup were offered the opportunity to participate. Of those, 29 T2DM patients (20 Jews and 9 Arabs), of whom 18 were men (14 Jews) and 11 were women (6 Jews), chose to take part in the study. Their mean age was 60.7 (*SD* = 8.17), with Jewish participants (*M* = 61.9, *SD* = 8.19) older on average than Arab ones (*M* = 58.1, *SD* = 7.90). Twenty-five participants (18 Jews) were married, two Arabs were widowed, and one Jewish participant each was divorced and single. We also interviewed two endocrinologists in an HMO clinic treating T2DM patients and two experts in eHealth, one a researcher in the field of T2DM and the other an employee in the digital services of the T2DM clinic. In addition, we explored a list of available digital tools for diabetes treatment with the aid of a diabetes HMO nurse. Finally, the resources provided to T2DM patients on the HMO website and on other public websites, including apps, were researched.

#### Instruments

##### Demographic questionnaire

The research team constructed two demographic questionnaires. The questionnaire for the T2DM patients included questions about age, gender, ethnicity, level of education, and year of diagnosis. It was constructed in Hebrew and translated to Arabic using back translation. In the initial phase, two native Arabic speakers, fluent in Hebrew, performed the translation. Subsequently, two educational counselors holding master’s degrees, who are native speakers of Arabic and speak Hebrew fluently, translated the questionnaire back from Arabic to Hebrew. The questionnaire items for the endocrinologists and technology experts inquired about age, gender, and years of expertise.

##### A semi-structured interview guide

The research team developed interview guides in Hebrew to inquire about digital tools used and available for T2DM patients’ self-management of the illness. To verify the suitability of the questions, the interview guide for patients with T2DM was first tested with two individuals who met the inclusion criteria, and changes were made accordingly. The guide was then translated into Arabic using the above-mentioned back translation method. Sample questions were: “Which HMO digital tools do you use to manage T2DM?” (patients); “What digital services are available at the HMO for diabetes treatment?” (endocrinologists); “What digital tools are available for diabetes treatment?” (eHealth experts). Follow-up questions inquired about each digital tool’s characteristics, purpose, and frequency of use.

#### Procedure

Following the approval of ethics from the college IRB (masked), T2DM patients who arrived at the diabetes HMO clinics were invited to participate in the study by the clinic nurse, and interested volunteers left their contact information. All study participants (including physicians and eHealth experts) signed an informed consent slip and filled out the demographic questionnaire. Phone interviews were conducted by two female research assistants (Jewish and Arab), graduate students in educational counseling, who each interviewed their respective participant group. Interviews lasted about 15 min and were recorded verbatim.

#### Data analysis

A manifest content analysis was applied to create a registry of digital tools available to and used by T2DM patients (43, 44). The content was analyzed using stages of decontextualization (e.g., identifying units of meaning), recontextualization (e.g., labeling similar units with a code), categorization (e.g., grouping similar codes into a category), and compilation (e.g., integrating the categories into themes and a coherent understanding of the topic). Interview data, the HMO website, and other public websites and apps were searched for types of digital tools provided and their purpose. These were tallied and grouped into codes, categories, and themes by type of tool and provider (HMO, non-HMO). A list of types of digital tools available from HMOs and non-HMOs was created and served as a basis for creating the DTUQ-D items.

#### Construction of the DTUQ-D

The identified types of digital tools available for patients’ self-management of T2DM were listed as questionnaire items (please see Appendix). Instructions were: “In the last 6 months, how often have you used the following tools for your diabetes management?” Possible responses were on a 4-point Likert scale ranging from 0 (*never*) to 3 (*regularly*). Five items were listed under HMO resources (e.g., a phone appointment with the physician), and five items were listed under non-HMO resources (e.g., websites providing relevant information for diabetes patients). In addition, to verify that no relevant tools had been overlooked, three additional open-ended items were added. Two read: “An additional tool that you are familiar with or use that was not mentioned” and provided space to add this information. The third item was included to verify that the six-month time frame did not exclude tools used annually; it read: “In the past year, have you used an additional tool (that was mentioned or not mentioned so far) to manage your diabetes? If so, what tool?” Thus, the questionnaire included 13 items overall.

To check for wording, accurateness, and clarity, the questionnaire was reviewed by an endocrinologist treating T2DM patients and was administered to two T2DM patients. Minor changes were made accordingly.

The questionnaire was then translated from Hebrew to Arabic by two experts proficient in both languages, with thorough attention to cultural sensitivity, ensuring the relevance and appropriateness of its content in the specific cultural context. Subsequently, it underwent a back translation from Arabic to Hebrew, facilitated by two educational counselors fluent in both languages. A few disagreements were discussed and resolved by the researchers, along with another research assistant proficient in Arabic and Hebrew. For international use, the questionnaire was also translated to English through a similar process, involving two native-speaking individuals fluent in both English and Hebrew.

### Phase 2: quantitative

The aim of this part of the study was to validate the newly developed Digital Tool Use Questionnaire for Diabetes (DTUQ-D). We assessed the reliability and validity of the questionnaire in both Hebrew and Arabic to establish its utility as a measurement tool for T2DM patients.

#### Participants

The sample was gathered via a survey company that ensures representation across all societal sections. Table 1 displays demographic characteristics of participants. There were 367 participants in total, 259 of whom were Jewish (70.6%) and 108 of whom were Arab (29.4%). Arab participants were Muslim (*n* = 75, 69.4%), Christian (*n* = 27, 25.0%), Muslim Bedouin (*n* = 4, 3.7%), and Druze (*n* = 2, 1.9%). About half the participants were male (around 55%), with a somewhat greater percentage of males among Arabs (about 64%) than Jews (about 51%). On average, participants were close to the age of 60, with Jews older (about 59) than their Arab counterparts (about 53). Jewish participants had higher levels of education than Arab participants. Diagnosis of the disease occurred up to 43 years ago, with an average of about 12 years, and with no ethnic difference.

**Table 1:** Demographic characteristics of participants (*N* = 367).

		Total	Arab	Jewish	
Gender *n* (%)	Male	202 (55.0)	69 (63.9)	133 (51.4)	*Z* = 2.20 (*p* = 0.028)
	Female	165 (45.0)	39 (36.1)	126 (48.6)	
Age *M* (*SD*) range		57.52 (10.26) 28–84	52.85 (10.44) 28–79	59.46 (9.55) 34–84	*t*(365)^(1)^ = 5.88 (*p* < 0.001)
Level of education *n* (%)	Less than high school	19 (5.2)	12 (11.1)	7 (2.7)	χ^2^(4) = 17.15 (*p* = 0.002)
	High school	68 (18.5)	26 (24.1)	42 (16.2)	
	Non-academic higher education	94 (25.6)	19 (17.6)	75 (29.0)	
	Bachelor’s degree	88 (24.0)	24 (22.2)	64 (24.7)	
	Master’s or Ph.D.	98 (26.7)	27 (25.0)	71 (27.4)	
Duration of T2DM *M* (*SD*) range		12.21 (9.62) 1–43	11.13 (8.04) 1–31	12.66 (10.19) 1–43	*t*(251.53)^(1)^ = 1.545.88 (*p* = 0.126)

^(1)^*t* for unequal variances.

#### Instruments

##### Demographic questionnaire

The inventory was constructed for the purpose of the current research. It included such demographic details as ethnicity, weight and height (BMI), access to mobile phone/Internet, and gender.

##### Digital tool use questionnaire for diabetes (DTUQ-D)

This questionnaire was constructed for the current study to examine the type, number, and frequency of digital tool use (for details, see Phase 1 above). Means were calculated for the purpose of the current study.

##### eHealth literacy scale (eHEALS)

The eHEALS (40) includes eight items dealing with awareness of health resources and the search for, utilization, and appraisal of health resources. Sample item: “I know how to find helpful health resources online.” Possible responses fall along a five-point Likert scale, ranging from 0 (*highly disagree*) to 4 (*highly agree*). The Hebrew version was found to be valid and reliable and is commonly used [e.g., (45)]. The questionnaire was translated to Arabic for the present study. It was first translated from Hebrew to Arabic by two experts proficient in both languages and then back translated from Arabic to Hebrew by two educational counselors fluent in the two languages. In the current study, principal components factor analysis yielded one factor explaining 76.72% of the variance (Eigenvalue = 6.14). Internal consistency for the whole sample was α = 0.96 (Jews: α = 0.95, Arabs: α = 0.96).

#### Procedure

The study followed the eHealth Code of Ethics (46). Upon obtaining approval from the college’s Ethics Committee (masked), the questionnaire was distributed by a survey company with an attached link. The survey company guaranteed a representative sample of Israeli society by adhering to key demographic criteria. Financial incentives were provided for participation, and demographic quotas were set before data collection began, ensuring a balanced and representative sample. Participants were informed of the voluntary nature of the study and their ability to terminate involvement at any point. We assured participants of the confidentiality of their responses, clarifying that the results would be used solely for research purposes. Patient data were anonymized in the collected questionnaires. Participants signed an informed consent form. Questionnaire completion time was approximately 10 min.

#### Data analysis

Data were analyzed using SPSS ver. 29. Descriptive statistics were employed for the demographic and background variables of the participants, with ethnic comparisons calculated by *t*-tests, Chi-squared tests, and *Z* ratios for the significance of the difference between independent proportions. Internal consistencies for eHEALS were calculated using Cronbach’s α. The DTUQ-D items were presented with frequencies and percentages, and ethnicity-based comparisons were performed using Chi-squared tests. To assess construct validity of the DTUQ-D, a multi-group confirmatory factor analysis (MGCFA) was conducted, utilizing AMOS software ver. 29. Fit measures, including CFI, NNFI, and RMSEA, were used to compare the unconstrained model, structural covariances model, and the measurement residuals model. Due to low fit, an exploratory principal axis factoring (EFA) with the criterion of Eigenvalue greater than 1 was calculated for the DTUQ-D by ethnicity. The total score was calculated using item means and compared across ethnic groups and genders with an analysis of variance. Pearson and Spearman correlations were calculated between DTUQ-D and eHEALS, as well as between DTUQ-D and the demographic variables.

Sample size was first calculated for a CFA, using Soper’s (47) online calculator. Using a moderate effect size of 0.30, power level of 0.80, three latent variables, and the 20 items of eHEALS and DTUQ-D, with α = 0.05, the minimum desired *N* is 323 participants. For a two-way ANOVA, with a moderate-low effect size of *f* = 0.15, α = 0.05, and a power level of 0.80, the minimum desired *N* is 351 [G*Power 3: (48, 49)].

The additional three open-ended items (11–13) of the DTUQ-D were not included in the statistical analysis but rather were assessed by content analysis post-administration. The responses of participants were reviewed and clustered into codes, categories, and themes of the digital tools mentioned.

## Results

The research aimed to assess the validity of the DTUQ-D to evaluate the type, number, and frequency of digital tools utilized by T2DM patients and to assess for variability among both genders and ethnic groups. The findings revealed that the digital tools used most often by Arab participants were the glucometer (about 63% used it often or regularly), websites with diabetes-related information (about 43%), the HMO website/app (to set an appointment, order medication, leave a message; about 31%), and diabetes management reminders (taking medication, making appointments, etc.; about 30%). The digital tools used most often by Jewish participants were the HMO website/app (to set an appointment, order medication, leave a message, etc.; about 80% used them often or regularly), the glucometer (about 69%), lifestyle apps (on a smart watch or mobile phone; about 41%), websites with diabetes-related information (about 38%), the HMO website/app (to get relevant information; about 35%), and diabetes management reminders (about 30%). That is, the extent of use of digital tools was generally higher among Jewish participants than among Arab participants. This trend applies to 6 of the 10 questionnaire items and is most notable regarding use of the HMO website and apps, as well as lifestyle apps (see Table 2).

**Table 2:** Distribution of DTUQ-D items by ethnicity (*N* = 367).

	Arab			Jewish			χ^2^(2)
	Never *n*(%)	Seldom *n*(%)	Often^(1)^ *n*(%)	Never *n*(%)	Seldom *n*(%)	Often^(1)^ *n*(%)	
HMO digital tools							
(1) Glucometer (at a subsidized fee)	32 (29.6)	8 (7.4)	68 (63.0)	40 (15.4)	40 (15.4)	179 (69.1)	12.01 (p = 0.002)
(2) Online training workshop with a nurse/nutritionist (at a subsidized fee)	78 (72.2)	12 (11.1)	18 (16.7)	175 (67.6)	54 (20.8)	30 (11.6)	5.76 (*p* = 0.056)
(3) Phone meeting with the physician	62 (57.4)	16 (14.8)	30 (27.8)	113 (43.6)	70 (27.0)	76 (29.3)	7.95 (p = 0.019)
(4) HMO website/app (to set an appointment, order medication, leave a message for a physician, etc.)	61 (56.5)	13 (12.0)	34 (31.5)	25 (9.7)	26 (10.0)	208 (80.3)	99.17 (p < 0.001)
(5) HMO website/app (to access information on diabetes, proper nutrition, physical activity, etc.)	71 (65.7)	15 (13.9)	22 (20.4)	95 (36.7)	73 (28.2)	91 (35.1)	26.12 (p < 0.001)
Other digital tools							
(6) Websites with T2DM-related information	44 (40.7)	18 (16.7)	46 (42.6)	74 (28.6)	86 (33.2)	99 (38.2)	11.23 (p = 0.004)
(7) Apps for monitoring/tracking glucose	68 (63.0)	13 (12.0)	27 (25.0)	169 (65.3)	38 (14.7)	52 (20.1)	1.30 (*p* = 0.522)
(8) Apps on a smart watch or mobile phone to promote a healthy lifestyle (e.g., counting steps or calories, recipes)	78 (72.2)	14 (13.0)	16 (14.8)	106 (40.9)	46 (17.8)	107 (41.3)	31.93 (p < 0.001)
(9) T2DM management reminders (e.g., taking or injecting medication, making appointments with a physician)	59 (54.6)	17 (15.7)	32 (29.6)	137 (52.9)	43 (16.6)	79 (30.5)	0.10 (*p* = 0.953)
(10) Individual or group support for T2DM on social networks (e.g., WhatsApp, Facebook, forums)	79 (73.1)	14 (13.0)	15 (13.9)	159 (61.4)	51 (19.7)	49 (18.9)	4.68 (*p* = 0.096)

^(1)^The DTUQ-D has a four-point scale: 0 = never, 1 = seldom, 2 = often, and 3 = regularly. As responses at level 3 (regularly) were rare, levels 2 and 3 (often and regularly) are combined.

Multi-group confirmatory factor analysis (MGCFA) was calculated for the 10 questionnaire items to evaluate the instrument’s construct validity. Items were divided into two factors, according to the initial definition of the questionnaire, and two groups were assessed: Jewish and Arab participants. Results showed that the unconstrained model had a good fit (CFI = 0.966, NNFI = 0.944, RMSEA = 0.036). Higher order comparisons showed low fit values (structural covariances model: CFI = 0.746, NNFI = 0.695, RMSEA = 0.083; measurement residuals model: CFI = 0.637, NNFI = 0.645, RMSEA = 0.090). The model was next assessed for one total factor, including all 10 items. The unconstrained model had a reasonable fit (CFI = 0.898, NNFI = 0.936, RMSEA = 0.061). Higher order comparisons showed low fit values (structural covariances model: CFI = 0.706, NNFI = 0.652, RMSEA = 0.089; measurement residuals model: CFI = 0.609, NNFI = 0.622, RMSEA = 0.092). Thus, the initial definition of the two factors of the questionnaire and its total score did not demonstrate construct validity across the two ethnic groups.

Based on these findings, an exploratory factor analysis (EFA) of the 10 DTUQ-D items was conducted separately for each ethnicity. Given that the correlations between the items ranged up to *r*s = 0.67 and *r*s = 0.40 among the Arab and Jewish sub-samples, respectively (*p* < 0.001), a principal axis factoring with oblique rotation was used. The criterion of Eigenvalue greater than one yielded three factors for each ethnicity, as shown in Table 3.

**Table 3:** Factor loadings for the DTUQ-D by ethnicity (*N* = 367).

	Arab			Jewish		
Factor	1	2	3	1	2	3
HMO digital tools						
(1) Glucometer (at a subsidized fee)	0.58	−0.04	0.10	0.14	−0.08	−0.33
(2) Online training workshop with a nurse/nutritionist (at a subsidized fee)	0.27	0.24	−0.32	−0.05	−0.60	0.02
(3) Phone meeting with the physician	0.22	0.18	−0.25	0.04	−0.47	−0.08
(4) HMO website/app (to set an appointment, order medication, leave a message for a physician, etc.)	−0.05	0.99	0.16	−0.03	−0.15	−0.77
(5) HMO website/app (to access information on diabetes, proper nutrition, physical activity, etc.)	0.02	0.70	−0.15	0.02	−0.57	−0.22
Other digital tools						
(6) Websites with T2DM-related information	0.84	0.02	0.02	0.49	−0.24	0.04
(7) Apps for monitoring/tracking glucose	0.59	0.01	−0.29	0.54	0.10	−0.06
(8) Apps on a smart watch or mobile phone to promote a healthy lifestyle (e.g., counting steps or calories, recipes)	0.30	−0.01	−0.56	0.58	0.02	−0.04
(9) T2DM management reminders (e.g., taking or injecting medication, making appointments with a physician)	0.58	0.12	−0.16	0.57	0.09	−0.11
(10) Individual or group support for T2DM on social networks (e.g., WhatsApp, Facebook, forums)	−0.12	−0.01	−0.95	0.44	−0.17	0.20
Eigenvalue	3.01	2.30	2.70	1.63	1.42	1.09
% of variance	38.56	10.54	6.92	20.55	9.09	4.93
Internal consistency	α = 0.81	*r* = 0.67 (*p* < 0.001)	α = 0.67	α = 0.66	α = 0.60	*r* = 0.37 (*p* < 0.001)

The three factors entail distinct combinations of items for each ethnicity, with low to good internal consistencies. In the Arab sector, the first factor involves information and monitoring tools, the second factor entails the HMO website and app, and the third factor includes online meetings. In the Jewish sector, the first factor comprises non-HMO digital tools, while the second and third factors entail specific digital tools offered by the HMO. Due to these ethnic variations in factor definitions, the total score for digital tool use was defined separately for each ethnic group (Arab sector: α = 0.84, Jewish sector: α = 0.69) and utilized for ethnic comparisons.

Table 4 presents *t*-values for eHEALS and DTUQ-D by ethnicity. The findings demonstrate notable ethnic disparities, indicating higher levels of both eHEALS and DTUQ-D scores among Jewish participants than Arab ones.

**Table 4:** Means, *SD*, and ranges of eHEALS and DTUQ-D by ethnicity (*N* = 367).

	Total *M* (*SD*)	Arab *M* (*SD*)	Jewish *M* (*SD*)	
eHEALS (0–4)	2.60 (1.02)	2.27 (1.13)	2.74 (0.95)	*t*(173.20)^(1)^ = 3.78 (*p* < 0.001) (*d* = 0.46)
DTUQ-D (0–3)	1.07 (0.61)	0.85 (0.69)	1.17 (0.54)	*t*(163.84)^(1)^ = 4.20 (*p* < 0.001) (*d* = 0.53)

^(1)^*t* for unequal variances.

Pearson and Spearman correlations were calculated to examine the associations between eHEALS and DTUQ-D, as well as between demographic variables and DTUQ-D, as presented in Table 5. Positive and significant correlations were found between eHEALS and DTUQ-D, indicating that higher eHEALS scores were associated with increased DTUQ-D scores and providing convergent validity to the newly developed questionnaire. Notably, demographic variables showed no significant associations with DTUQ-D. It is noteworthy that gender differences in DTUQ-D scores were not significant, males: *M* = 1.08, *SD* = 0.64, females: *M* = 1.07, *SD* = 0.56, *t*(363.54) = 0.24, *p* = 0.810; nor was the interaction between ethnicity and gender significant, *F*(1, 363) = 1.57, *p* = 0.210, η^2^ = 0.004. Gender differences in eHEALS scores were also non-significant, males: *M* = 2.51, *SD* = 1.10, females: *M* = 2.71, *SD* = 0.92, *t* (364.77) = 1.84, *p* = 0.066; as was the interaction between ethnicity and gender, *F*(1, 363) = 0.01, *p* = 0.941, η^2^ = 0.001.

**Table 5:** Pearson and Spearman correlations for DTUQ-D with eHEALS and demographic variables (*N* = 367).

	eHEALS^(1)^	Age^(1)^	Duration of T2DM^(1)^	Level of education^(2)^
DTUQ-D				
Total	0.30^***^	0.03	−0.01	0.05
Arab	0.33^***^	0.01	−0.05	0.14
Jewish	0.22^***^	−0.06	−0.02	−0.04

^(1)^Pearson correlations; ^(2)^Spearman correlations; ^***^p < 0.001.

### Post-administration adjustment of DTUQ-D

Based on the analysis of the three open-ended questions, an additional item was added to the questionnaire in retrospect, “Other digital tools (such as Excel for monitoring, blood pressure monitor, digital scale).” This item, number 11, addresses digital tools that were not included in the original version but rather were mentioned by the participants in the validation sample. We recommend adding this item to the questionnaire.

## Discussion

This study aimed to develop and assess the effectiveness of the DTUQ-D as a screening tool for identifying the type, number, and frequency of digital tools used by T2DM patients for self-management of the illness, both within HMOs, online, and through apps. This investigation holds significant importance given rapid technological advancements in recent years, which have led to substantial changes characterized by digital, efficient, and cost-effective management of T2DM [e.g., (2)]. In the first phase our research questions addressed the digital tools utilized by and available to T2DM patients for self-management of their illness. Based on interviews we generated the questionnaire items.

An additional objective was to assess the tool’s validity among individuals with T2DM, including both Jewish and Arab populations in Israel and looking at both genders. The main research hypothesis regarding the validity and reliability of the constructed tool was corroborated. On the whole the results indicate the questionnaire can effectively identify digital tools used by T2DM patients of both genders, despite some differences in factor structures between the two ethnic groups.

The first hypothesis, suggesting a positive correlation between DTUQ-D and eHEALS scores, was supported, confirming the convergent validity of the questionnaire. Moreover, a reasonable internal reliability was observed among Jewish T2DM patients and a good internal reliability among Arab T2DM patients. This finding holds dual significance. First, it indicates that the newly developed questionnaire assesses the digital tools used by T2DM patients and gauges the extent of their usage. Second, it is reasonable to assume that individuals possessing elevated eHealth literacy levels are more inclined to actively utilize websites and applications that offer self-management treatments for T2DM.

In addition, a factor analysis was conducted to assess the construct validity of the DTUQ-D. This analysis was conducted separately for Jewish and Arab participants, in line with the second hypothesis suggesting potential cultural differences in the identified factors of the DTUQ-D. The hypothesis, which suggested lower digital tool usage by Arabs, was subsequently confirmed.

The analysis for Arab participants yielded three distinct factors. The first included: (item 1) “glucometer” from the first scale of HMO Digital Tools, and three items from the second scale of Other Digital Tools, i.e., digital medical services not provided by the HMO, namely: (item 6) “websites with T2DM-related information,” (item 7) “apps for monitoring/tracking glucose,” and (item 9) “T2DM management reminders (e.g., taking or injecting medication, making appointments with a physician).” The second factor consisted of two items related to services provided by the HMO: (item 4) “HMO website/app (to set an appointment, order medication, leave a message for a physician, etc.)” and (item 5) “HMO website/app (to access information on diabetes, proper nutrition, physical activity, etc.).” Finally, the third factor included two items from the HMO Digital Tools subscale: (item 2) “online training workshop with a nurse/nutritionist” and (item 3) “phone meeting with the physician,” as well as two items from the Other Digital Tools subscale: (item 8) “apps on a smart watch or mobile phone to promote a healthy lifestyle (e.g., counting steps or calories, recipes),” and (item 10) “individual or group support for T2DM on social networks (e.g., WhatsApp, Facebook, forums).”

The first factor found for Arabs encompasses digital tools that can be frequently used or easily accessed at home, including websites with T2DM-related information, apps for monitoring/tracking glucose, and T2DM self-management reminders. The focus on the glucometer in the first factor could indicate a specific interest or reliance on this tool for self-management, given its convenience for use at home. The second factor focuses specifically on services the HMO offers, such as setting appointments, ordering medication, and accessing relevant information through the HMO website/app. The third factor involves online interaction with professionals (nurse, nutritionist, and physician) and participation in T2DM online support on social networks and lifestyle apps. Notably, the services in this last factor were those used most infrequently by Arab participants.

Three factors were also identified for Jewish T2DM patients. The first factor included all five items in the Other Digital Tools subscale (items 6–10; see above for details of each item). This factor suggests that non-HMO tools are used similarly by Jewish participants and consist of tools that can be accessed at home. The second factor included several items from the HMO Digital Tools subscale – items 2, 3, and 5. This second factor primarily consists of information provided by online interaction with professionals and via the HMO website and are services that were moderately to infrequently used. Finally, the third factor included the rest of the items in the HMO Digital Tools scale (items 1 and 4), which are related to the most frequently used tools and services provided by the HMO.

In line with the second hypothesis, some variations can be identified between Jewish and Arab T2DM patients in their preferences and usage patterns of digital tools related to T2DM self-management. Based on the provided information, Jewish patients appear to have a more pronounced focus on HMO services. Nonetheless, there are differences in the frequency of use of these services, with the third factor tools the most frequently used (glucometer and HMO website to set appointments, etc.). Although overall, Arab T2DM patients use digital tools less than Jewish patients, they show a more diverse range of digital tool usage, including both HMO tools and lifestyle and self-management components that are not HMO-provided.

Cultural variations in perceptions of healthcare providers and trust in health systems suggest that Arab T2DM patients in Israel are likely to exhibit lower levels of trust in the medical system, potentially diminishing the adoption of digital services provided by the HMO. Indeed, research conducted in Israel within the Arab minority population revealed them to have significantly lower levels of trust in the healthcare system than Israeli Jews, which impacts adherence to public health recommendations (50).

Another possible explanation for the observed ethnic differences is limited access to web infrastructure and lower eHealth literacy in Arab society in Israel. The digital disparity between the Jewish majority and Arab minority is notably apparent in terms of Internet access and usage patterns (51). Furthermore, the distinctive characteristics of the Arab minority, including their socio-demographic status and the conservative nature of the culture, may contribute to the digital divide (30, 32). Understanding these distinctions can be valuable in tailoring T2DM self-management interventions to better suit the needs and preferences of each community. For example, HMOs can provide training in digital tool usage for T2DM self-management geared to each population group. For groups with low eHealth literacy, such training can be initially face-to-face while practicing online interaction later. Further research and qualitative exploration could provide deeper insights into the specific reasons behind these observed differences, allowing for more targeted and culturally sensitive healthcare interventions.

### Limitations

The current study is subject to a few limitations. Firstly, the sample comprised a total of 367 participants: 259 Jews and 108 Arabs. Although the sample size of the Jewish T2DM patients was adequate, the smaller number of Arab participants could produce less stable results, such that findings for the Arab sample should be considered preliminary. Secondly, given the specific characteristics of the ethnic groups studied in the current research, caution is advised in generalizing the study findings to a broader population, as there is a potential for Type I error, stemming from multiple comparisons. It is strongly recommended that future research replicate this study with a larger sample size and explore group differences by cultural background, gender, and age. This will help validate the findings and evaluate psychometric properties of the instrument. Thirdly, the study is based on a cross-sectional research design limiting the results’ generalizability. In future research it is strongly recommended to further examine this issue using in-depth interviews or longitudinal studies. Thus, it will be possible to delve into the internet usage patterns of each group in relation to T2DM. Such an approach will aid in uncovering the factors influencing internet usage rates among various groups, including the Arab population in Israel. Finally, the questionnaire was primarily tailored for application within a specific cultural framework, thus restricting its generalizability to other cultural or linguistic contexts. It is strongly recommend to further examine its effectiveness in international settings.

### Contributions

This study has theoretical, methodological, and practical implications. The results contribute to the theoretical understanding of digital tool use among Israeli Jewish and Arab individuals with T2DM. By identifying distinct factors and patterns, our study may enrich the theoretical framework in the field of health behavior and technology adoption. In addition, the research lays the groundwork for cross-cultural comparisons between different populations in the context of T2DM self-management. This comparative approach can deepen insights into the factors influencing digital tool use across diverse cultural settings.

In terms of methodological advancements, we developed an innovative questionnaire through a combination of qualitative and quantitative methods, proposing a foundation for a construction method that other researchers can adopt. In addition, the research introduces and assesses the DTUQ-D, providing a valuable screening tool for studying digital tool utilization in the context of T2DM self-management. This questionnaire could serve as a foundation for future research, offering a standardized and systematic approach to assess digital tool usage. Researchers can build upon the identified factors and explore additional dimensions, thereby enhancing understanding of the evolving role of digital tools in healthcare, particularly in the context of T2DM self-management. Furthermore, the questionnaire can be easily modified, by adjusting the instructions and some of the specific T2DM items, to assess digital tool use by patients with other illnesses.

The questionnaire can be scored and coded in several ways, depending upon the research question. It can provide researchers with information about the number, frequency, and types of digital tools used. For example, in a study examining the number of digital tools used, the *number* of items that received responses in the range of 1 to 3 (*seldom* to *regularly*) should be counted. When the research question examines the frequency of digital tool use, we recommend calculating a *total* score (based on the Likert scale values) that reflects accumulated tool use. It is also possible to calculate a *mean* score, rendering a frequency score across all items, as done in the current study. This latter approach takes into account individual differences in digital use that can be related, for instance, to the severity of the illness or the inclination to use digital tools. Lastly, to gauge the *type* of tools used, it is possible to calculate the sum of items within each factor.

From a practical point of view, the information gleaned from the DTUQ-D can assist in developing targeted interventions or guidelines to optimize the use of digital technologies while reducing potentially problematic usage patterns (52). Thus, our findings have practical implications for healthcare practitioners, policymakers, and digital health intervention developers. Comprehending the specific digital tools favored by Jewish and Arab populations can contribute to the development of such targeted interventions, enhancing the efficacy of T2DM self-management strategies. Moreover, by tailoring digital tools and interventions to the preferences and needs of specific cultural groups, it is possible to enhance engagement in and the effectiveness of the support provided by HMOs.

In sum, the contributions of the research lie in advancing theoretical understanding, providing a methodological tool, and offering practical insights that can inform healthcare practices and the development of culturally sensitive digital health interventions. This knowledge can facilitate more effective communication and collaboration between healthcare professionals and patients in managing T2DM.

## Data availability statement

The raw data supporting the conclusions of this article will be made available by the authors, without undue reservation.

## Ethics statement

The studies involving humans were approved by the college’s Ethics Committee. The studies were conducted in accordance with the local legislation and institutional requirements. The participants provided their written informed consent to participate in this study.

## Author contributions

OP: Conceptualization, Data curation, Funding acquisition, Investigation, Methodology, Project administration, Resources, Supervision, Visualization, Writing – original draft, Writing – review & editing. EH: Conceptualization, Funding acquisition, Investigation, Resources, Visualization, Writing – review & editing. MB-N: Conceptualization, Investigation, Resources, Visualization, Writing – original draft, Writing – review & editing.

## Appendix

**Table:**

DTUQ-D					
Over the last 6 months, to what degree have you utilized the following tools for managing your diabetes?					
		0 Never	1 Seldom	2 Often	3 Regularly
HMO digital tools					
1	Glucometer (at a subsidized fee)				
2	Online training workshop with a nurse/nutritionist (at a subsidized fee)				
3	Phone meeting with the physician				
4	HMO website/app (to set an appointment, order medication, leave a message for a physician, etc.)				
5	HMO website/app (to access information on diabetes, proper nutrition, physical activity, etc.)				
Other digital tools					
6	Websites with T2DM-related information				
7	Apps for monitoring/tracking glucose				
8	Apps on a smart watch or mobile phone to promote a healthy lifestyle (e.g., counting steps or calories, recipes)				
9	T2DM management reminders (e.g., taking or injecting medication, making appointments with a physician)				
10	Individual or group support for T2DM on social networks (e.g., WhatsApp, Facebook, forums)				
